# Impact of Membrane Pore Size on the Clarification Performance of Grape Marc Extract by Microfiltration

**DOI:** 10.3390/membranes9110146

**Published:** 2019-11-06

**Authors:** Francisca Mora, Karla Pérez, Carolina Quezada, Carla Herrera, Alfredo Cassano, René Ruby-Figueroa

**Affiliations:** 1 Department of Chemistry, Universidad Tecnológica Metropolitana, Las Palmeras 3360, Ñuñoa, Santiago 7800003, Chile; francisca.moras@utem.cl; 2 Programa Institucional de Fomento a la Investigación, Desarrollo e Innovación, Universidad Tecnológica Metropolitana, Ignacio Valdivieso 2409, San Joaquín, Santiago 8940577, Chile; karla.perezr@utem.cl (K.P.); carolina.quezadab@utem.cl (C.Q.); cherrera@utem.cl (C.H.); 3 Institute on Membrane Technology, ITM-CNR, c/o University of Calabria, via P. Bucci, 17/C, I-87030 Rende, Italy; a.cassano@itm.cnr.it

**Keywords:** grape marc, clarification, microfiltration, fouling analysis

## Abstract

The influence of membrane pore size on the permeate flux, fouling mechanism, and rejection of soluble and suspended solids, as well as of phenolics and anthocyanins, in the clarification of grape marc extract by microfiltration (MF) was studied. MF was operated by using three monotubular ceramic membranes with a pore size of 0.14, 0.2, and 0.8 µm, respectively, according to a batch concentration configuration in selected operating conditions (2.25 bar as operating pressure, 4.93 L/min as feed flow rate, and 25 °C as operating temperature). No significant differences in the permeate flux values were appreciated despite the difference in pore size. The mathematical analyses of the flux behavior revealed that intermediate pore blocking is the predominant mechanism for 0.14 and 0.2 µm membranes, whereas complete pore blocking prevails for the 0.8 µm membrane. Differences in the fouling mechanism were associated with differences in the total phenols rejection: the highest rejection was observed for the 0.8 µm membrane followed by 0.2 and 0.14 µm membranes. All selected membranes showed low rejection of sugars, with values lower than 10%, and no retention towards anthocyanins. All the clarified extracts showed a turbidity lower than 4.87 NTU. Based on the experimental results, the 0.14 µm membrane appeared as the best option for the clarification of grape marc extract.

## 1. Introduction

These days, the globe is facing critical challenges in terms of environmental issues, which are moving several industries to the development of sustainable processes for every kind of production. In this regard, the industries are greatly interested in implementing innovative, productive cycles through the sustainable use of resources and the implementation of clean production patterns, which all concepts of circular economy based on the three R΄s, Reduce, Reuse and Recycle [1]. In this context, biorefinery development is considered an appropriated strategy for the proper use of renewable sources. In particular, wastes generated from agro-food processes are considered renewable feedstock enriched by bio-based compounds, which can be used as a raw material for the formulation of pharmaceutical, cosmetic, or food products [2,3].

Within the agro-industrial sector, grape cultivation, mainly set aside for wine production, is one of the most extensive activities. In 2018, 44.35 million tons of grape were destined for wine production with a total production of 292 MhL [4]. It has been estimated that 18–20% of the grapes used for winemaking remain as a solid residue known as grape marc (or pomace), which includes grape skin, pulp, seeds, stems, and residual juice [5]. Grape marc is characterized by high values of chemical oxygen demand (COD) and biological oxygen demand (BOD); therefore, it can be easily digested by microorganisms generating greenhouse gases like methane. In this way, the global system enables the generation of power from grape marc, thus reducing the power requirements of wineries and minimizing the negative impact on the environment [6].

Grape marc and seeds are also a valuable source of phenolic compounds, and the most abundant are anthocyanins, hydroxybenzoic and hydroxycinnamic acids, flavan-3-ols, flavonols, and stilbenes [3,7,8]. These compounds are widely recognized for their high antioxidant activity [9]. As such, they are capable of scavenging a wide range of reactive oxygen, nitrogen, and chlorine species, such as superoxide anion, hydroxyl radical, peroxyl radicals, hypochlorous acid, and peroxynitrous acid. According to the evidence, the oxidative damage is associated with the development of most major age-related degenerative diseases; thus, it has been speculated that phenolic compounds may have protective effects against such conditions decreasing the risk for cardiovascular diseases, neurodegenerative disorders, and type 2 diabetes [10,11,12], as well as anti-inflammatory, antiviral, and antimicrobial effects [13,14]. In this regard, these compounds exhibit therapeutic and health-promoting effects and can be exploited as functional ingredients for the food, pharmaceutical, and cosmetic industries [15]. As a consequence, the recovery of biophenols from grape marc has gained increasing attention worldwide [16,17].

Several processes have been proposed for the extraction of phenolic compounds from grape marc including both conventional and non-conventional methodologies. Traditional techniques, such as solid–liquid or Soxhlet extractions, are based on the use of ethanolic or methanolic solutions as solvents [15,18]. These methods are characterized by several disadvantages, including long extraction times, loss of compounds due to hydrolysis and oxidation during extraction, and potential environmental pollution due to large volumes of organic solvent. Therefore, they have been gradually replaced by novel extraction methods aimed at increasing extraction yields while preserving the extract quality, and at reducing energy consumption and operating time. In this regard, techniques such as ultrasound-assisted extraction (UAE) [19], microwave-assisted extraction [20,21], subcritical assisted extraction [22], pulsed electrical field [23], and supercritical fluid extraction [24] have been applied successfully.

Grape marc extracts are heterogeneous suspensions comprised of particles of the submicrometer to millimeter-scale including macromolecules (i.e., polysaccharides) and haze-forming components (suspended solids, colloidal particles, proteins, and polyphenols). Conventional methods for the production of clarified extracts involve centrifugation, filtration on plates, diatomaceous earth filtration, and fining agents such as proteins, bentonites, gelatin, and silica hydrogel. These processes are generally slow, and in addition, the use of fining aids is characterized by different drawbacks such as the risks of dust inhalation with consequent health problems due to handling and disposal, adsorption of target compounds, environmental problems, and significant costs [25]. The use of cross-flow microfiltration (MF) as an alternative greatly simplifies the process operation and result in an increase in juice yield, improved product quality and avoidance of filtering aids. MF membranes have demonstrated their efficiency in the clarification of several fruit juices and fermented beverages [26,27,28], allowing the ability to remove particles, plant cellular debris, and microorganisms (yeasts, fungi, spores, and bacteria) in the range of the micrometer scale (0.1–10 μm). As for other pressure-driven membrane processes, MF offers significant advantages over conventional methodologies, which include the possibility to operate at low-moderate temperatures, no use of chemical agents, and easy scale-up and low energy consumption.

The major challenge limiting the performance of MF membranes is membrane fouling, which is defined as a long term flux decline caused by the deposition of feed compounds on the membrane surface or within membrane pores. It is a key factor affecting the economic and commercial viability of a membrane process, which essentially depends on the obtained permeate fluxes [29].

Process parameters and feed composition, as well as the chemical and morphological structure of the membrane material, play a crucial role in fouling. In particular, the membrane pore size has a direct relation with the type of fouling that is predominant and, consequently, with the retention of specific compounds [30]. It has been reported that membranes with smaller pore sizes are more resistant to fouling [31], while other studies have opposite results [32]. The feed composition should also be taken into account to evaluate the effect of pore size on membrane fouling. Depending on feed composition, fouling mechanisms can occur either by partial, total, or internal pore blocking (if feed particles are comparable in size or smaller than membrane pore size) or by cake formation, which is due to the deposition of larger particles onto the membrane surface [33].

In view of the foregoing, this work aimed at evaluating the effect of membrane pore size on the permeate flux and the retention of phenolic compounds, sugars, and turbidity in the clarification of grape marc extract with ceramic MF membranes. An analysis of fouling was also performed by using the models proposed by Field et al. [34] and Yee et al. [35] in order to describe the dynamics of the flux decay for the proposed membranes in selected operating conditions, establishing the fouling occurrences.

## 2. Materials and Methods

### 2.1. Grape Marc Extract

**Figure 1:**
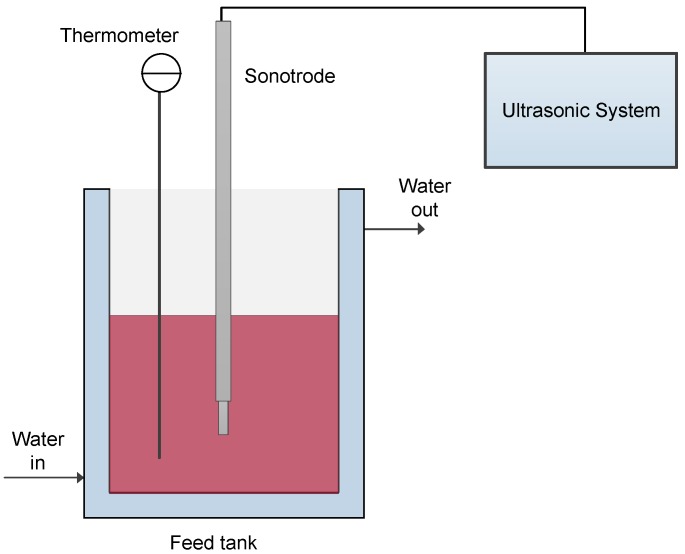
Schematic diagram of the experimental set-up for ultrasound-assisted extraction.

Grape marc from red grape (Carménère variety) was supplied by Concha y Toro winemaker (Pencahue, Chile) and stored at −80 °C until use. The ultrasound-assisted extraction (UAE) was carried out in an ultrasonic bath system (SFX550 Sonifier, Branson Ultrasonics Corporation, Danbury, CT, USA) in optimal operating conditions: grape marc, 23.85% w/w; ethanol, 40% w/w; water, 36.15% w/w; amplitude, 20%; temperature, 22 °C; operating time, 15 min. The experimental setup for the extraction process is illustrated in Figure 1. The total extracted solution (10 L), in sequential steps, was filtered on nylon cloth and stored at −5 °C until its characterization and clarification by MF.

### 2.2. MF Set-Up and Procedure

**Figure 2:**
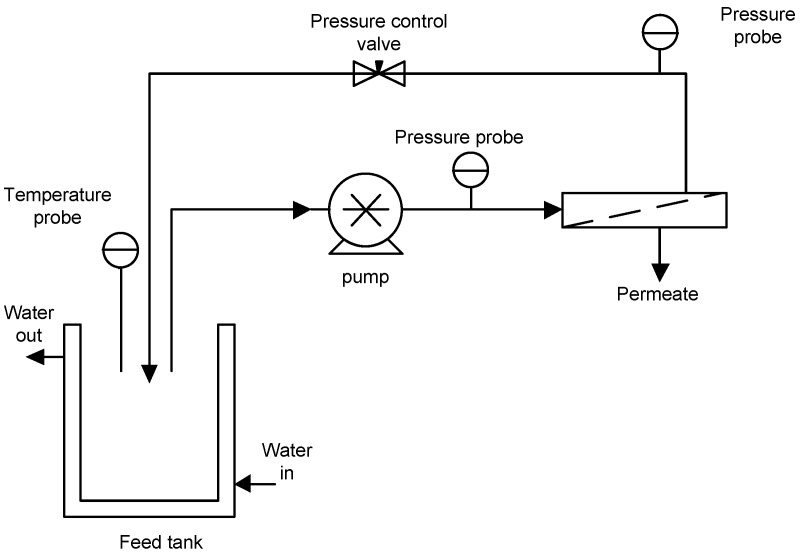
Schematic diagram of Microfiltration experimental set-up.

MF experiments were performed by using a laboratory unit (Figure 2) equipped with a positive displacement rotary vane pump (Fluid-o-Tech^®^, PO1011, Milano, Italy) and a stainless steel housing able to accommodate a tubular membrane module with a length of 250 mm and an effective membrane area of 0.005 m^2^. The feed temperature was adjusted by circulating tap water in a two-layered feed tank.

Three different mono-tubular ceramic membranes (Tami Industries, Nyons, France) with pore sizes of 0.14, 0.2, and 0.8 µm were tested. These membranes showed initial hydraulic permeabilities of 0.57, 0.63, and 1.32 L/m^2^ bar, respectively.

The MF system was operated at a transmembrane pressure (TMP) of 2.25 bar, a temperature of 25 °C, and an axial feed flow rate (Q_f_) of 4.93 L/min according to the batch concentration configuration in which the permeate was collected separately, while the retentate was continuously recycled back to the feed tank. The permeate was collected for 3 h, and the permeate flux was calculated using the following equation:

(1) $$J=\frac{V}{At}$$

where *A* is the membrane area, *V* is the collected permeate volume, and *t* the operating time.

### 2.3. Membrane Fouling Analysis

#### 2.3.1. Hermia’s Model Modified for Cross-Flow Filtration

Hermia [36] developed four empirical models for dead-end filtration at constant pressure corresponding to four basic types of fouling: complete blocking, intermediate blocking, standard blocking, and cake layer formation. The type of fouling considered depends on the value of the parameter *n* in Equation (2):

(2) $$\frac{d^{2}t}{dV^{2}}=\beta {\left(\frac{dt}{dV}\right)}^{n}$$

where *V* is the cumulative volume of filtrate, *t* the operating time, and *β* a constant.

These models were modified by Field et al. [34], which proposed a mathematical model to describe the fouling mechanisms during the crossflow MF of passion fruit juice. The characteristic equation proposed by the model is described in Equation (3):

(3) $$\frac{-dJ}{dt}=K\left(J-J_{ss}\right)*J^{2-n}$$

where *J_ss_* is the steady-state flux, and *K* is a constant whose dimension depends on the values of *n*; *n* is a general index that, depending on the fouling mechanism, assumes different values as reported in Table 1 [37].

The complete pore blocking model (n = 2) assumes that the size of the solute particles is greater than that of the membrane pores. Therefore, it occurs on the membrane surface rather than inside membrane pores. When particles are smaller than the pores, they enter the membrane pores, thereby reducing the pore volume. This mechanism of fouling is named as standard pore blocking (n = 1.5). The intermediate pore blocking (n = 1) assumes that some particles can obstruct the pore entrance but not completely block it. Finally, in the cake filtration (n = 0), particles that do not enter the pores form a cake layer on the membrane surface. Consequently, the overall resistance is composed of a cake resistance and a membrane resistance, which is assumed to remain unchanged.

#### 2.3.2. Unified Model of Flux Decline

Yee et al. [35] developed a unified model to describe the time dependence of flux decline over the long-term operation of whey during ultrafiltration. The proposed model consisted of three piecewise exponential decay models corresponding to three stages of fouling, namely concentration polarization, protein deposition, and consolidation of the deposits. The general form of the model is shown in Equation (8):

(8) $$q_{p}\left(t\right)=q_{p,f,\infty }+k_{f}\exp\left(b_{f}t\right)$$

where the subscript *f* (1, 2, 3) represents one of the three stages of fouling: *f* = 1 for concentration polarization, *f* = 2 for deposition, and *f* = 3 for long-term fouling or consolidation; $q_{p,f,\infty }$ is the steady-state permeate flow rate achieved for each stage of fouling; b_f_ (1, 2, 3) are the rate constants for the decrease in permeate flow rate; and *k_f_* is the exponential factor for each stage of fouling (L/min). The authors reported that the permeate flux is controlled by concentration polarization in the first step of filtration (5–6 min), followed by protein deposition until approximately 3 h have passed [35]. Then the decrease in permeate flux is dominated by a long-term fouling mechanism. 

### 2.4. Analytical Measurements

#### 2.4.1. Anthocyanins Analysis

The identification of anthocyanins in the UAE extract was performed by HPLC-DAD-ESI-MS/MS. The HPLC-DAD conditions were: C18 column (250 mm × 4.6 mm, 5 μm), oven temperature 40 °C, injection volume 50 μL, mobile phase flow 0.5 mL/min, mobile phase composition water/acetonitrile/formic acid (87:3:10% v/v/v) (solvent A), and water/acetonitrile/formic acid (40:50:10% v/v/v) (solvent B). The ESI-MS/MS parameters were: positive ionization mode, 200–1200 m/z range, 4000 V of ionization voltage, the capillary temperature at 450 °C, nebulizer gas 40 psi, and auxiliary gas 50 psi.

The analyses of the anthocyanins in the UAE grape marc extract and clarified solutions were performed with a PerkinElmer AltusTM A-30 ultra-performance liquid chromatography UPLC^®^ System (PerkinElmer Inc., Waltham, MA, USA) equipped with an A-30 Quaternary Solvent Delivery Module, A-30 Sampling Module, A-30 PDA detector, A-30 FL detector, and A-30 RI detector. The separation was carried out by using a Waters ACCQ-TAGTM ULTRA C18 column (2.1 mm × 100 mm, 1.7 µm). The chromatographic conditions were carried out according to Ruiz et al. [38] with slight modifications. The transformation of the HPLC gradient to UPLC gradient was made using the Columns Calculator software (Waters Corporation, Milford, MA, USA). The mobile phase was constituted by water/acetonitrile/formic acid (87:3:10% v/v/v) (solvent A) and water/acetonitrile/formic acid (40:50:10% v/v/v) (solvent B). The flow rate was 0.5 mL min^−1^, the column temperature 40 °C, and the injection volume was 2.0 μL. The gradient program was as follow: from 6 to 30% of B in 2 min, from 30 to 40% of B in 3 min, up to 60% of B in 0.5 min, and followed by 2.5 min of stabilization at 6% of B. The total run was 8 min. The detection was at 520 nm, and for quantification, a malvidin-3-glucoside external calibration curve was used. Results were expressed as malvidin-3-glucoside equivalents.

#### 2.4.2. Total Phenolic Compound Analysis

Total phenolic compounds (TPC) were estimated colorimetrically by using the Folin–Ciocalteau method [39], which is based on the reduction of tungstate and/or molybdate in the Folin–Ciocalteau reagent by phenols in an alkaline medium resulting in a blue-colored solution. Results were expressed as mg gallic acid equivalents per g of sample dry weight (mg GAE/g). The absorbance measurements were performed by using an UV/VIS/NIR Lambda 750 spectrometer (PerkinElmer Inc., Waltham, MA, USA) at 765 nm.

#### 2.4.3. Soluble and Suspended Solids Measurements

The suspended solids measurement was performed by using a turbidimeter (Hanna Instruments Inc., USA) at 20 °C. Results were expressed as Nephelometric Turbidity Unit (NTU). On the other hand, the soluble solids content was determined at 20 °C by using a hand refractometer (Atago Co., Ltd., Tokyo, Japan) with a scale range of 0–32 °Brix. Results were expressed as °Brix.

### 2.5. Data Analysis

Results of the physicochemical analysis of the grape marc extract and related samples obtained in the clarification step were expressed as mean ± standard deviation (SD) of three replicates. The adjustment of the experimental data to the fouling model was performed by a least squares fitting procedure.

Determination of the quality of fit was evaluated using the root mean square error (RMSE) and the percentage of variability explained (R^2^) at a 95% confidence level. On the other hand, the validation procedure of the fouling mechanism was performed employing the residual analysis, which should come from a normal distribution. Shapiro–Wilks (S-W) and Kolmogorov–Smirnov (K-S) tests were used for this purpose. Statistic and mathematical computations were performed in Statgraphics Centurion XVI (Statgraphics Technologies, The Plains, VA, USA) and Excel 2010 (Microsoft, Redmond, WA, USA).

## 3. Results and Discussion

### 3.1. UAE Extract Characterization 

**Figure 3:**
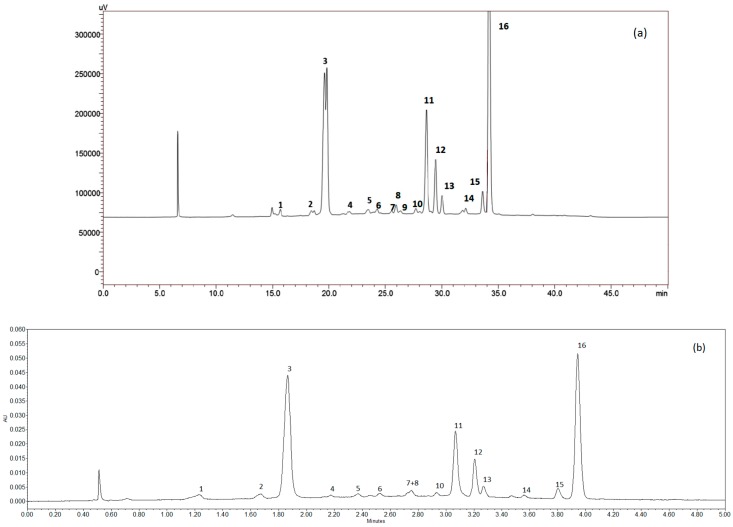
Chromatograms of the characterization of grape marc extracts obtained from an ultrasound assisted extraction (UAE). (**a**) HPLC-DAD-ESI-MS/MS analysis; (**b**) ultra-performance liquid chromatography (UPLC) analysis.

Figure 3a shows the chromatogram of the HPLC-DAD-ESI-MS/MS analyses in which 16 peaks associated with phenolic compounds were detected, and 15 compounds were identified, as shown in Table 2. The majority of the compounds are anthocyanins (13), whereas two of them were identified as flavonols. These compounds are related to the distinctive characteristic of Carménère grapes regarding astringency and color [40,41]. Malvidin 3-(coumaroyl)-glucoside was the most representative compound followed by malvidin-3-glucoside, and malvidin 3-(acetyl)-glucoside.

Figure 3b shows the chromatogram obtained by UPLC in which the 16 peaks, observed in HPLC-DAD-ESI-MS/MS, can be appreciated. In particular, the elution pattern was the same; however, the time required for the analysis was appreciably lower. In addition, the extract showed the value of the total phenols at 4.25 ± 1.87 mg GAE/g dry weight, the soluble solids content at 18.64 ± 1.11 °Brix, and the suspended solids content at 640.44 ± 380.32 NTU.

### 3.2. Permeate Flux and Membrane Fouling Analysis

**Figure 4:**
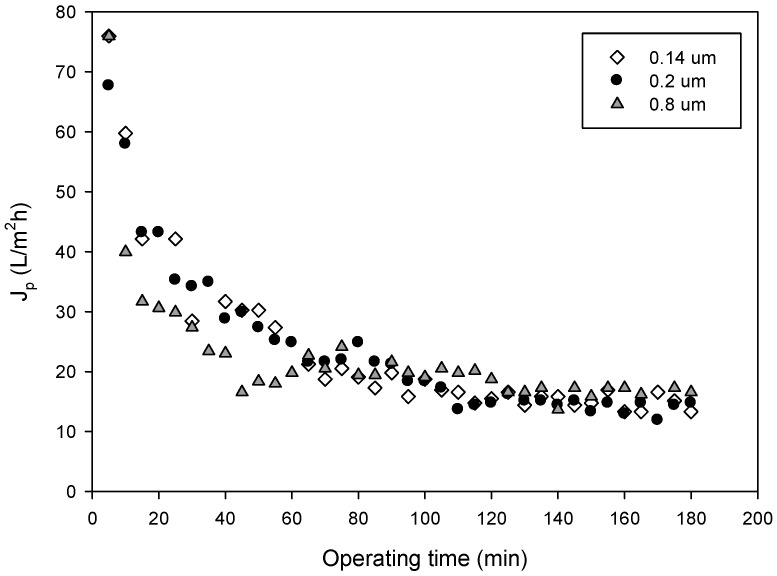
Time course of permeate flux for the investigated membranes. Operating conditions: TMP, 2.25 bar; Q_f_, 4.93 L/min; T, 25 °C.

Figure 4 shows the time course of the permeate flux measured for the different membranes in selected operating conditions. No significant differences in the permeate flux (F-ratio: 0.11; p = 0.8948) were observed despite the fact that membrane pore sizes are different. These results have a direct correlation with the type of fouling predominant for each membrane, which produces similar values of permeate flux at a pseudo-steady-state. Permeate fluxes resulted in the same order of those obtained by Prodanov et al. [42] in the clarification of grape pomace extracts using polysulphone hollow fiber membranes with a pore size of 0.22 μm and molecular weight cut-off of 500 kDa.

Table 3 shows the results of the fitted parameter for the modified Hermia model for cross-flow filtration [34] and the unified model for flux decline described by Yee et al. [35]. The determination of the predominant fouling mechanism for each membrane is based on the criteria of the maximum R^2^ and the minimum value of RMSE. Besides, it is expected that residuals (the difference of observed and estimated values for each model) should have random behavior because that is an indication that the models have not presented autocorrelation. In this regard, the intermediate blocking was the type of fouling with a better degree of fitting for membranes with a pore size of 0.14 and 0.2 µm, and with R^2^ values of 95.2 and 97.8%, respectively. In addition, for both membranes, the RMSE was lower than 0.0035. On the other hand, for the membrane with a pore size of 0.8 µm, the membrane fouling followed the complete pore blocking model, which gave the best goodness of fit based on a higher R^2^ and a lower RMSE. These results clearly indicated a strong correlation between the pore size and the fouling mechanism, which in turn affects the membrane performance.

According to data reported in Table 3, the unified model for flux decline showed higher values of R^2^ and lower RMSE for the three membranes studied. Additionally, two of the membranes (0.2 and 0.8 µm) present random residuals; thus, the model is validated for those membranes.

**Figure 5:**
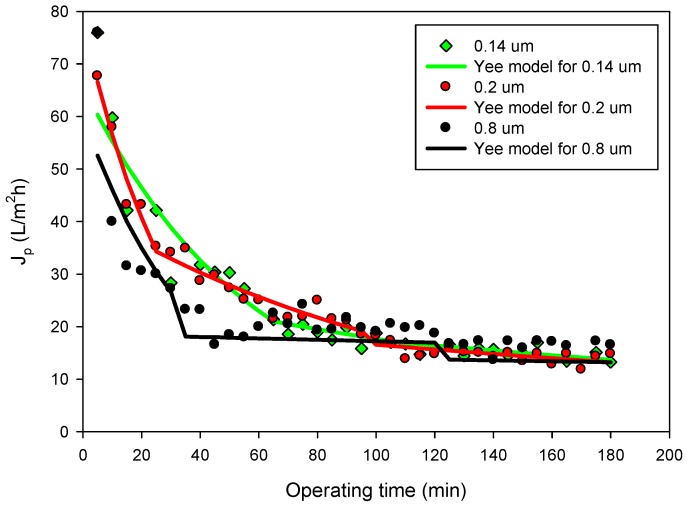
Experimental results and predicted values by the Yee model for the studied membranes.

The fit of the unified model to the experimental data is demonstrated in Figure 5, which shows the experimental and the predicted values of permeate flux for all tested membranes when the process is operated in the selected operating conditions. In addition to the absolute value of permeate flux, fouling dynamics can affect the dynamic behavior of the grape marc extract MF process. For the membrane with a pore size of 0.14 µm, there is an initial concentration polarization until 65 min of the process, followed by a deposition until 180 min. On the other hand, the 0.2 µm membrane showed a concentration polarization until 25 min, followed by a deposition until 100 min and a final long-term fouling. The membrane with a pore size of 0.8 µm showed a concentration polarization until 35 min, followed by deposition until 125 min and posterior long term fouling. The dynamic mechanism of fouling, described by Yee et al. [35], is more realistic in a complex system like a membrane process. The membrane with a pore size of 0.14 µm was characterized by a longer concentration polarization time. This phenomenon had a direct relation with membrane recovery and the rejection of valuable compounds.

### 3.3. Quality of Microfiltered Grape Marc Extract

**Figure 6:**
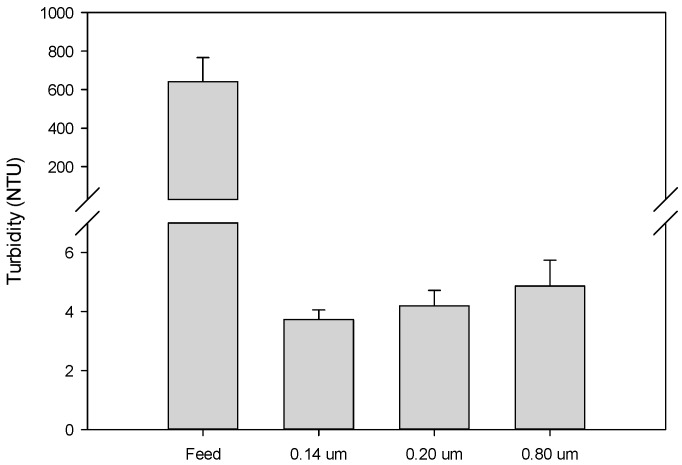
Suspended solids content in the grape marc extract (feed) and the permeate stream of investigated membranes.

As shown in Figure 6, suspended solids (turbidity) in the grape marc extract were almost removed completely and the turbidity of clarified extract was in the range 3.8–4.8 NTU, depending on the membrane pore size. For all selected membranes, the turbidity rejections were higher than 99%. Similar results were obtained by Prodanov et al. [42] in the clarification of grape pomace extracts with 500 kDa and 0.22 μm polysulphone membranes in hollow fiber and spiral-wound configurations, respectively. Similarly, fly-ash-based ceramic membranes with pore diameters of 0.30 μm and 1.25 μm effectively resulted in the clarification of kiwifruit juice completely removing the suspended solids from the fresh juice. On the other hand, the use of larger pore sizes (of about 2.13 µm) produced lower rejections of suspended solids (of about 45%) and low quality clarified juice [43].

**Figure 7:**
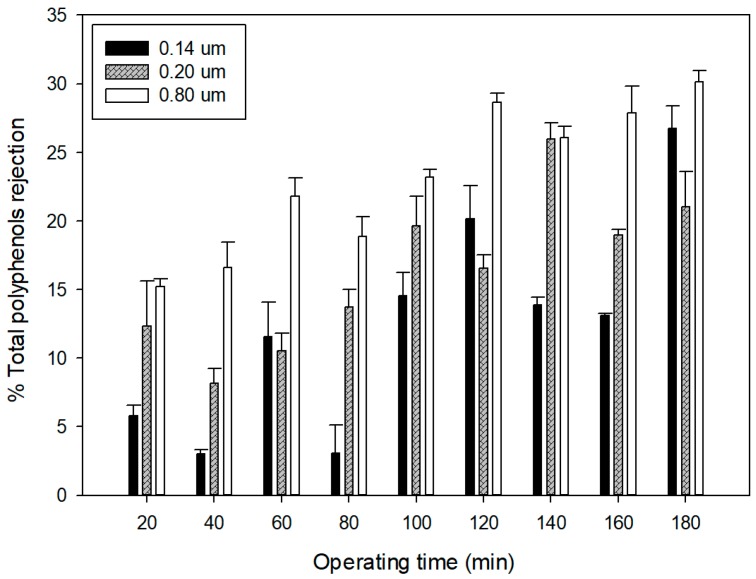
Rejection of MF membranes towards total phenols.

The observed rejections of selected membranes towards total phenolic compounds showed significant differences (F-ratio: 6.47; p = 0.0056) (Figure 7). In particular, the membrane with a pore size of 0.8 μm showed higher rejection values in comparison with the other two membranes. For this membrane, the data of fouling analyses indicated a complete pore blocking as the best fitting model to represent the flux decline mechanism; on the other hand, the intermediate pore blocking, was the prevailing mechanism affecting the 0.14 and 0.2 μm membranes (see Section 3.2). The lowest rejection was observed for the 0.14 μm membrane, with an average value of 12.42%. These results are in agreement with those reported by Jin et al. [31], which compared the fouling characteristics of different pore-sized (from 80 to 300 nm) submerged ceramic membranes. Results indicated that membranes with the roughest surface and biggest pore size (300 nm) had the highest fouling potential. Similarly, the blocking index of the 0.4 μm membranes had larger results than that of the 0.2 μm membrane under the same filtration pressure and filtration flux due to more severe membrane blocking [44].

**Figure 8:**
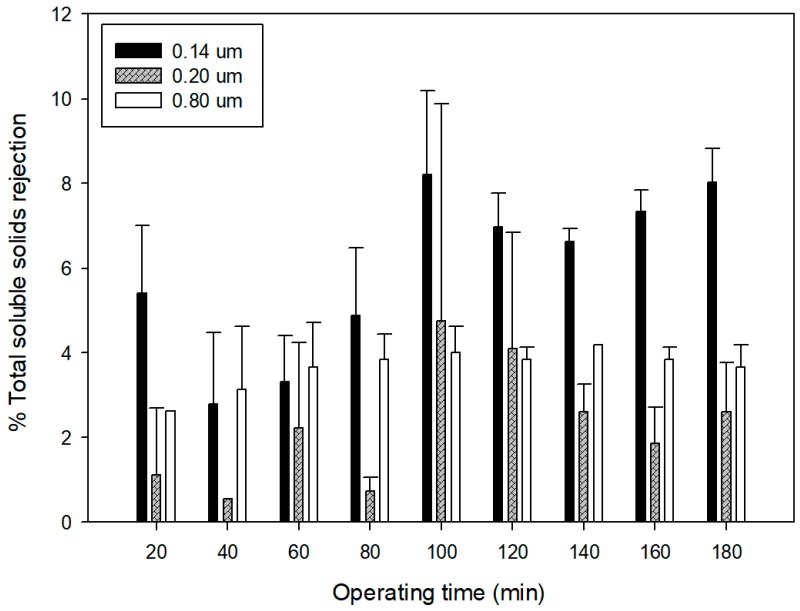
Rejection of MF membranes towards soluble solids content.

Significant differences (F-ratio: 19.34; p = 0.000) between the selected membranes were also observed for the rejection towards soluble solids (Figure 8). In this case, the 0.14 μm membrane showed the highest rejection, in agreement with its lowest pore size, and the predominant fouling mechanism detected as intermediate pore blocking. This type of fouling acts as an additional barrier for the transfer of compounds with lower molecular weight (i.e., sugars) to the permeate stream. The increased rejection as a function of the operating time can be attributed to the increased concentration of retained solutes on the feed side according to the batch concentration configuration, which in turn results in a more severe concentration polarization.

For all membranes rejection values that resulted lower than 10%, a total soluble solid content in the clarified extract higher than 17.5 °Brix was assured. Higher retention of soluble solids (23%) was measured by Razi et al. [45] in the clarification of tomato juice with polyvinylidenefluoride membranes in a flat sheet configuration and with a pore size of 0.45 μm.

In Table 4, the content of specific anthocyanins in the clarified grape marc extract of each membrane is reported. The experimental results indicate that these compounds are well preserved in the permeate stream, independently of the fouling mechanisms observed for each one of the investigated membranes. The observed rejection for these compounds was in the range 1.8–4.7% for the 0.14 μm membrane, 1.8–6% for the 0.2 μm membrane, and 2.9–9.7% for the 0.8 μm membrane, respectively. These results were in agreement with the rejection data measured by Prodanov et al. [42] in the clarification of marc grape pomace with 0.2 and 0.6 μm polysulphone membranes.

## 4. Conclusions

Three ceramic MF membranes with different pore sizes (0.14, 0.2, and 0.8 µm) have been used for the clarification of a grape marc extract obtained by ultrasound-assisted extraction. No significant differences in the permeate flux were observed in the selected operating conditions despite the fact that membrane pore sizes are different.

The analyses of the fouling mechanism according to Hermia’s models adapted for cross-flow MF revealed that intermediate pore blocking was the predominant mechanism for 0.14 and 0.2 µm membranes, whereas complete pore blocking prevailed for the 0.8 µm membrane. Experimental results obtained from the lab-scale rig over 180 min of operation have provided evidence to support the presence of three stages of fouling (concentration polarization, solute deposition, and consolidation of deposits) according to a unified model that describes the time dependence of flux decline. Based on this model, the membrane with a pore size of 0.14 µm was characterized by a longer concentration polarization time. This phenomenon is strongly correlated with the recovery and/or rejection of valuable compounds.

Differences in the fouling mechanism were associated with differences in the total phenols rejection; the lowest rejection was observed for the 0.14 μm membrane, with an average value of 12.42%. All the membranes showed low rejection of sugars, with values lower than 10%, and no rejection towards the main anthocyanins were detected. In all the cases, suspended solids in the grape marc extract were almost removed completely, and the turbidity of the permeate was lower than 4.87 NTU.

On the basis of experimental results, the 0.14 µm membrane appeared as the best option for the production of clarified anthocyanin-rich grape marc extracts of interest for innovative formulations in the food, nutraceutical, and pharmaceutical industry. Further investigations related to the effect of operating conditions on the membrane performance, long-term experiences with higher membrane area, and the correlation of permeate flux with a phenomenological or stochastic model are needed in order to address design criteria for industrial application.

## Figures and Tables

**Table 1:** Summary of characteristic equations for constant pressure filtration laws.

Function	n Value	Model	Equation
Complete pore blocking	n = 2	$J=\left(J_{o}-J_{ss}\right)exp\left(-K_{c}t\right)+J_{ss}$	(4)
Standard pore blocking	n = 1.5	$J={\left(J_{o}^{-0.5}+K_{s}t\right)}^{-2}$	(5)
Intermediate pore blocking	n = 1	$J=\frac{J_{ss}}{\left[1-\left(\frac{J_{o-}J_{ss}}{J_{o}}\right)\exp\left(-J_{ss}K_{i}t\right)\right]}$	(6)
Cake formation	n = 0	$K_{g}t=\frac{1}{J_{\mathrm{ss}}^{2}}\left[\ln\left(\frac{J}{J_{o}}*\frac{J_{o}-J_{ss}}{J-J_{ss}}\right)-J_{ss}*\left(\frac{1}{J}-\frac{1}{J_{o}}\right)\right]$	(7)

**Table 2:** Liquid chromatography-mass spectrometry LC-MS data of phenolic compounds from the extract obtained by UAE.

Peak	Compound	tR (min)	λ (nm)	[M-H]+	Product Ions
1	Petunidin-3-glucoside	16.11	280, 523	479	317, 302, 274
2	Peonidin-3-glucoside	18.62	280, 327, 520	463	301, 286
3	Malvidin-3-O-glucoside **	20.15	280, 350, 528	493	331, 315, 287
4	Quercetin-3-glucuronide ***	21.94	250, 353	479	303
5	Quercetin 3-glucoside ***	22.75	250, 351	465	303
6	Petunidin 3-(acetyl)-glucoside	24.55	280, 530	521	317, 302
7	Petunidin 3-(caffeoyl)-glucoside	25.83	280, 530	641	317, 302, 274
8	Delphinidin 3-rutinoside	26.20	280, 320, 529	611	303
9	n.i. *	26.63	276, 500	559	355, 339, 311
10	Peonidin 3-(acetyl)-glucoside	27.70	280, 312,520	505	301, 286
11	Malvidin 3-(acetyl)-glucoside	28.90	276, 529	535	331, 315, 287
12	Malvidin 3-(caffeoyl)-glucoside	29.71	281, 327, 530	655	331, 315, 287
13	Petunidin 3-(coumaroyl)-glucoside	30.30	281, 532	625	317, 302
14	Malvidin-3-rutinoside	32.40	280, 530	639	331, 315, 287
15	Peonidin 3-(coumaroyl)-glucoside	33.85	283, 310, 523	609	301, 286
16	Malvidin 3-(coumaroyl)-glucoside	34.40	282, 532	639	331, 315, 287

* n.i., not identified. ** signal dissociation, presents identical mass spectrometry data corresponding to the same compound. *** Flavonols.

**Table 3:** Fitted parameters for fouling models according to the modified Hermia’s models for cross-flow filtration, unified model of flux decline, and statistical model analysis for the studied membranes. S-W: Shapiro–Wilks test; K-S: Kolmogorov–Smirnov test; RMSE: root mean square error.

Fouling Model			Membrane Pore Size		
			0.14 µm	0.2 µm	0.8 µm
Hermia’s models adapted for cross-flow microfiltration [34]	Complete pore blocking	Kc	32.535	26.001	95.9345
		R^2^	95.2028	96.3153	91.6064
		RMSE	0.0049	0.0017	0.0038
		S-W test (*p*-value)	7.83 × 10^−10^	0.2746	4.3 × 10^−6^
		K-S test (*p*-value)	0.000657	0.6340	0.0846100
	Standard pore blocking	Ki	0.656384	0.73382	0,67193
		R^2^	67.13958	55.6330	89.655
		RMSE	0.0042	0.0013	0.0028
		S-W test (*p*-value)	1.43 × 10^−3^	0.0157	2.89 × 10^−9^
		K-S test (*p*-value)	0.022328	0.4564	0.006085
	Intermediate pore blocking	Ks	36.9425	47.5505	129.562
		R^2^	95.2028	97.8559	90.5891
		RMSE	0.0035	0.0008	0.0023
		S-W test (*p*-value)	9.49 × 10^−10^	0.0003	5.95 × 10^−9^
		K-S test (*p*-value)	0.000516	0.2808	0.037512
	Cake formation	Kg	5,800,344	8,642,697	11,030,594
		R^2^	88.53165	97.5383	82.30814
		RMSE	0.0031	0.0013	0.0015
		S-W test (*p*-value)	2.89 × 10^−8^	3.76 × 10^−6^	3.74 × 10^−11^
		K-S test (*p*-value)	0.004099	0.33078	0.0104003
Unified model for flux decline Yee et al. [35]		K1	0.000292	0.00054	4.55 × 10^−4^
		b1	−10.9087	−10.734	−10,9964
		K2	0.0000811	0.00013	1.291 × 10^−5^
		b2	−11.7368	−11.3531	−12.1731
		K3	5.05 × 10^−5^	4.7 × 10^−5^	1.115× 10^−5^
		b3	−11.9331	−12.0056	−12.3943
		R^2^	90.594	97.35	80.576
		RMSE	3.3 × 10^−3^	0.5 × 10^−3^	1.6 × 10^−2^
		S-W test (*p*-value)	5.86 × 10^−10^	0.61	1.13 × 10^−6^
		K-S test (*p*-value)	9.41 × 10^−5^	0.7652	0.04760

**Table 4:** Anthocyanins content in the feed and permeate stream of investigated membranes.

Compound	Sample	Membrane Pore Size		
		0.14 µm	0.2 µm	0.8 µm
Malvidin-3-O-glucoside (mg/L)	Feed	43.77 ± 0.58	43.29 ± 2.1	40.62 ± 1.8
	Permeate *	42.95 ± 0.71	42.72 ± 1.26	39.44 ± 0.22
Malvidin 3-(acetyl)-glucoside (mg/L) **	Feed	18.81 ± 4.8	19.13 ± 1.3	17.49 ± 2.6
	Permeate *	18.01 ± 0.39	18.08 ± 0.30	16.76 ± 0.12
Malvidin 3-(coumaroyl)-glucoside **	Feed	79.46 ± 4.7	79.72 ± 5.1	76.10 ± 3.2
	Permeate *	75.71 ± 2.18	74.91 ± 1.60	68.65 ± 12.85

* The value reported is the mean ± SD for the samples taken during 180 min of filtration (9 samples taken every 20 min). ** malvidin 3-(acetyl)-glucoside and malvidin 3-(coumaroyl)-glucoside were expressed as malvidin-3-glucoside equivalents.
